# Selenium enrichment of broccoli sprout extract increases chemosensitivity and apoptosis of LNCaP prostate cancer cells

**DOI:** 10.1186/1471-2407-9-414

**Published:** 2009-11-30

**Authors:** Rizky Abdulah, Ahmad Faried, Kenji Kobayashi, Chiho Yamazaki, Eka W Suradji, Kazuto Ito, Kazuhiro Suzuki, Masami Murakami, Hiroyuki Kuwano, Hiroshi Koyama

**Affiliations:** 1 Department of Public Health, Gunma University Graduate School of Medicine, Japan; 2 Department of General Surgical Science (Surgery I), Gunma University Graduate School of Medicine, Japan; 3 Department of Urology, Gunma University Graduate School of Medicine, Japan; 4 Department of Clinical Laboratory Medicine, Gunma University Graduate School of Medicine, Japan; 5 Faculty of Pharmacy, Padjadjaran University, Indonesia; 6 Faculty of Medicine, Padjadjaran University, Indonesia

## Abstract

**Background:**

Broccoli is a Brassica vegetable that is believed to possess chemopreventive properties. Selenium also shows promise as an anticancer agent. Thus, selenium enrichment of broccoli has the potential to enhance the anticancer properties of broccoli sprouts.

**Method:**

Selenium-enriched broccoli sprouts were prepared using a sodium selenite solution. Their anticancer properties were evaluated in human prostate cancer cell lines and compared with those of a control broccoli sprout extract.

**Results:**

Selenium-enriched broccoli sprouts were superior to normal broccoli sprouts in inhibiting cell proliferation, decreasing prostate-specific antigen secretion, and inducing apoptosis of prostate cancer cells. Furthermore, selenium-enriched broccoli sprouts but, not normal broccoli sprouts, induced a downregulation of the survival Akt/mTOR pathway.

**Conclusion:**

Our results suggest that selenium-enriched broccoli sprouts could potentially be used as an alternative selenium source for prostate cancer prevention and therapy.

## Background

Selenium (Se) is essential for humans and has been reported to play a beneficial role in the prevention of cardiovascular disease [1-3] and cancer [4-6]. The main Se source for humans comes from diet; however, the Se concentration in foods varies widely and is dependent on the Se concentration of the soil in which the food was grown. People living in Se-poor areas have a very low Se intake, which is often associated with Se-deficiency-related diseases, such as Keshan disease, Kashin-Beck disease and cancer [5,7-9].

The generation of various Se-enriched foods would be beneficial to prevent Se deficiency in people [10,11]. Scientists have reported the benefits of enriching broccoli with Se for cancer prevention [12-14]. Broccoli is known to contain sulforaphane, an active anticancer agent [15,16]. As a member of the Se-accumulator Brassica family, broccoli accumulates Se-methylselenocysteine as the major Se compound when it is germinated in Se-enriched media [17,18]. Interestingly, Se-methylselenocysteine is also known to possess cancer-protective properties [19,20]. Therefore, Se-enriched broccoli accumulates two active anticancer agents: sulforaphane and Se-methylselenocysteine.

Recently, broccoli sprouts have received considerable attention, because they contain ten times more sulforaphane than broccoli florets [21,22]. Broccoli sprouts have also been reported to inhibit cancer growth *in vitro *and *in vivo *[23-25]. Moreover, epidemiological studies have shown that both cruciferous vegetables [26] and selenium [4] may reduce the incidence of prostate cancer. Thus, in the present study, we investigated the antiproliferative activity of a control broccoli sprout extract (CSp) and a Se-enriched broccoli sprout extract (SeSp). We also explored the underlying mechanism of action in the survival and apoptosis pathways in human prostate cancer cell lines. Our results suggest that SeSp acts as a promising Se source for prostate cancer prevention and therapy.

## Methods

### Reagents and standards

All reagents used for total Se, Se speciation, and sulforaphane analysis were of analytical grade and were purchased from Wako Pure Chemical (Tokyo, Japan) unless stated otherwise. For the preparation of solutions and sample treatment, Ultrapure Milli-Q water (Millipore, Tokyo, Japan) was used. Cyclohexane and 2,3-diaminonaphthalene, used for total Se determination, were purchased from Dojindo Laboratories (Tokyo, Japan). Se standards, sodium selenate, selenomethionine, and Se-methylselenocysteine were purchased from Wako Pure Chemical (Tokyo, Japan); sodium selenite was purchased from Sigma Chemical (MO, USA) and selenocystine was purchased from Acros Organic (Geel, Belgium). Sulforaphane standard was purchased from LKT Laboratories (MN, USA).

### Broccoli sprout extract preparation

SeSp was prepared using the method described by Sugihara et al. [27]. Briefly, broccoli sprout seeds (Tohoku Co., Ltd., Ibaraki, Japan) were germinated in 10 ppm of a sodium selenite solution to produce SeSp or in deionized water to produce CSp. The total germination period was 7 days. The sprouts were then harvested and kept at -80°C until extraction.

For Se speciation and cell culture treatment, 500 mg of broccoli sprouts (wet weight) was extracted in 1 ml of deionized water by sonication (Branson Sonifier 450, CT, USA) for 1 minute on ice. After centrifugation at 2,500 rpm for 10 minutes, the supernatant was filtered through a 0.45- μm membrane filter (Toyo Roshi, Tokyo, Japan). The extract was prepared on the same day as the Se speciation analysis or cell culture treatment.

### Determination of total Se concentration

The total Se content of broccoli sprouts was measured by a method described previously [28] using a Twinkle LB970 spectrofluorometer (Berthold Technologies, Bad Wildbad, Germany) at an excitation wavelength of 378 nm and an emission wavelength of 525 nm. The accuracy of this analysis was monitored by the measurement of bovine liver SRM 1577b as the reference material (National Institute of Standards and Technology, MD, USA).

### Se speciation analysis by HPLC/ICP-MS

Se speciation was measured using high-performance liquid chromatography (HPLC) coupled to inductively coupled plasma mass spectrometry (ICP-MS). Fifty microliters of extract was injected into an Asahipak GS-320 HQ size exclusion column (Showa Denko, Tokyo, Japan) and detected by an ICP-MS (Perkin-Elmer Sciex ELAN 6100, Ontario, Canada) connected to a GemTip™ cross-flow nebulizer with a Scott-type spray chamber. All ICP-MS operating parameters were adjusted to obtain maximum signal intensity. The instrumental operating conditions are given in Additional File 1.

### Sulforaphane analysis by HPLC

Sulforaphane extraction and analysis were performed by slight modification of the method described by Liang et al. [29]. Briefly, 500 mg of broccoli sprouts (wet weight) was homogenized by sonication in 1 ml of de-ionized water for 1 minute on ice. After incubation at room temperature for 30 minutes, the homogenate was extracted three times with 5 ml of methylene chloride. The methylene chloride fractions were combined and salted with 1 g of anhydrous sodium sulfate. The methylene chloride fraction was then dried at 30°C using a rotary evaporator (Eyela Rotary Vacuum Evaporator N-N series, Tokyo, Japan). The residue was dissolved in acetonitrile and stored at -80°C until analysis.

Analysis of sulforaphane was performed via the standard addition method. Sulforaphane standards of 10, 20, and 40 μg/ml in acetonitrile were mixed with the sulforaphane extract at a ratio of 1:1 (v:v). Fifty microliters of this mixed sample was then applied to an HPLC system (Waters, MA, USA). Separation was performed by an Inertsil ODS-3 column 4.6 × 250 mm (GL Sciences, Tokyo, Japan) with 70% acetonitrile as the mobile phase at a flow rate of 1 ml/min. Sulforaphane was detected at a UV absorbance of 254 nm.

### Cell culture and treatment

Three human prostate cancer cell lines (LNCaP, PC-3 and DU-145) were purchased from Dainippon Pharmaceutical (Tokyo, Japan). One non-cancerous cell line (CHEK-1, an immortalized human esophageal cell line) was provided by Dr. H. Matsubara. CHEK-1 was established by transduction of human papillomavirus type 16 E6/E7 into primary cultures of esophageal keratinocytes [30]. The cell lines were cultured in RPMI-1640 medium (Sigma, MO, USA) supplemented with 10% fetal bovine serum and antibiotics (100 U/ml penicillin and 100 μg/ml streptomycin).

For cell treatments, various concentrations of CSp and SeSp extracts were added to the cell culture medium, followed by the addition of L-Methioninase (Wako, VA, USA) in order for SeSp to generate methylselenol. After 24 hours, cells were released from the CSp or SeSp treatment, the medium was replaced, and cells were harvested at the indicated time.

### Drug sensitivity assay

Cell proliferation analysis was performed with cells in the presence of various concentrations of broccoli sprout extracts by a colorimetric methyl thiazolyl tetrazolium (MTT) assay, as described previously [31]. Briefly, cells (2 × 10^4 ^in 50 μl/well) were plated in 96-well plates. After the initial cell seeding, different concentrations of broccoli sprout extracts were added and incubated for 24 hours. Ten microliters of WST-8 assay cell-counting solution (Dojindo Lab., Tokyo, Japan) was added to each well and incubated at 37°C for 3 hours. After the addition of 100 μl/well of 1 N HCl, the cell proliferation rate was then determined by measuring the absorbance at a wavelength of 450 nm with a reference wavelength of 650 nm. The absorbance was read using a microtiter plate reader (Becton-Dickinson, NJ, USA). Results were derived from triplicate experiments.

### Prostate-Specific Antigen (PSA) analysis

At approximately 80% confluence, the cells were treated with the IC_50 _of each sprout extract. After 24 hours, the cell medium was collected, dead cells and debris were removed by centrifugation, and the clear supernatant fraction was stored at -80°C until analysis of PSA secretion. PSA values were determined at the Gunma University Hospital Laboratory by an immunoenzymatic assay using an ST AIA-PACK PSA II kit (Tosoh Bioscience, Tokyo, Japan), following the method described by the manufacturer. PSA values were normalized to the total protein concentration of viable cells, as described elsewhere [32], using a ND-1000 Spectrophotometer (NanoDrop Technology, DE, USA).

### Flow cytometric analysis

Cell sample preparation and propidium iodide staining were performed as described previously [31]. At around 80% confluency, the cells were treated with the IC_50 _of each sprout extract. For cell cycle distribution experiments, living cells were harvested at 0 (untreated), 24, 48, and 72 hours and analyzed using a FACScan Coulter EPICS XL Flow Cytometer (Beckman Coulter, CA, USA).

### Microscopic examination

Cells were cultured and treated with CSp and SeSp as described above. Morphological changes were examined at the time indicated and photographed using a regular phase-contrast microscope.

### Cell extraction and Western blot analysis

Protein concentrations were determined using a BCA Protein Assay Kit (Pierce, IL, USA). Protein (40 μg) was electrophoresed on 5-20% Tris-Tricine ReadyGel (Bio-Rad, Tokyo, Japan), and electro-transferred to a hybond-enhanced chemiluminescence membrane (Amersham, Buckinghamshire, UK). Apoptosis-related proteins were analyzed using caspase-3, caspase-8, caspase-9, and PARP antibodies in 1:1000 dilutions (Cell Signaling Technology, MA, USA). Survival-related proteins were analyzed using antibodies against total Akt, phospho-Akt (p-Akt), total mTOR, and phospho-mTOR (p-mTOR) at 1:1000 dilutions (Cell Signaling Technology, MA, USA). Protein bands were detected using an enhanced chemiluminescence detection system (Amersham, Buckinghamshire, UK). β-actin (Sigma) served as the loading control. Scanning densitometry was performed with Adobe Photoshop (Apple, Inc., CA, USA), and analyzed with Quantity One (Bio-Rad, Tokyo, Japan).

### Statistical analysis

Results of the total Se and sulforaphane concentrations were compared using paired t-tests. A two-way ANOVA was also used to analyze the difference of the effect of CSp and SeSp to PARP, p-Akt and p-mTOR protein expression. All statistical analyses were performed by R statistical software [33].

## Results

### Total Se determination, Se speciation, and sulforaphane concentration analysis

Total Se analysis showed that the 10 ppm sodium selenite solution used to germinate the broccoli sprout seeds was able to increase the Se concentration of the sprouts. The total Se concentration of SeSp was 10.24 μg/g, which was significantly higher than that of CSp at 0.01 μg/g of Se (Additional File 2).

**Figure 1:**
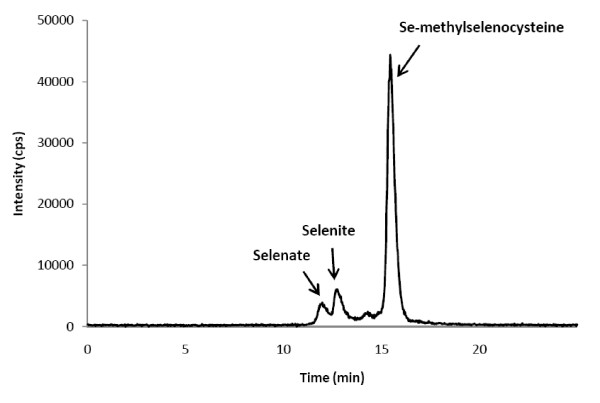
**Selenium speciation of the SeSp extract using HPLC/ICP-MS**. cps: counts per second.

A chromatogram of the Se speciation of SeSp is shown in Figure 1. The retention time of the major Se compound of the extract was compared with that of an authentic selenium compound, such as selenite, selenate, Se-methylselenocysteine, selenomethionine, or selenocystine. The retention time of Se in the SeSp extract was matched to the retention time of the selenate, selenite, and Se-methylselenocysteine standards at 12.2, 12.9, and 15.7 minutes, respectively (Figure 1). By contrast, there was no Se peak detected in the CSp extract (data not shown).

The concentration of selenate, selenite, and Se-methylselenocysteine was then calculated by measuring the large area below each corresponding peak, which was then applied to the standard curve made using the standard at several concentrations. The concentrations of selenate, selenite, and Se-methylselenocysteine in the SeSp extract were 2 μM, 6.16 μM, and 24.2 μM, respectively (Additional File 2).

The sulforaphane analysis showed that the differences in sulforaphane concentration of CSp (375.87 ± 17.98 μM) and SeSp (316.53 ± 43.54 μM) were not statistically significant (Additional File 2).

### Drug sensitivity assay

**Figure 2:**
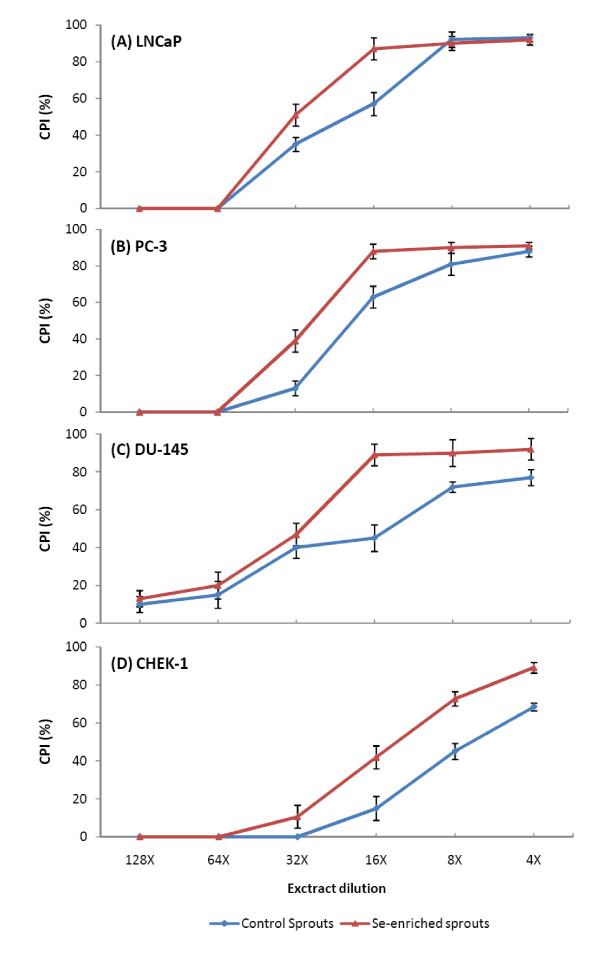
**Effects of 24 h treatment of various CSp and SeSp concentrations to (A) LNCaP, (B) PC-3, (C) DU-145 and (D) CHEK-1 cells viability**. CPI: cell proliferation inhibition.

As shown in Figure 2, treatment with both extracts resulted in the dose-dependent inhibition of prostate cancer cell growth when assessed at 24 hours post-treatment. In LNCaP cells, the IC_50 _value of the CSp extract was at 19-times dilution, while that of the SeSp extract was at 32-times dilution. In PC-3 cells, the IC_50 _value of the CSp extract was at 20-times dilution, and that of the SeSp extract was at 28-times dilution. In DU-145 cells, the IC_50 _value of the CSp extract was at 15-times dilution, while that of the SeSp extract was at 30-times dilution.

For the non-cancerous CHEK-1 cell lines, higher concentrations of both sprout extracts were required to inhibit cell growth. The IC_50 _value of the CSp extract on CHEK-1 cells was at 7-times dilution, while that of the SeSp extract was at 14-times dilution. Based on these data, it is tempting to speculate that SeSp might be less harmful to non-cancerous cells. The concentration of sulforaphane and Se-methylselenocysteine at the IC_50 _dilution of CSp and SeSp extracts on LNCaP, PC-3, DU-145 and CHEK-1 cells are shown in Additional File 3.

PC-3 cells demonstrated the highest sensitivity to treatment with CSp and LNCaP cells were most sensitive to treatment with SeSp. On the other hand, DU-145 cells demonstrated the lowest sensitivity to CSp treatment and PC-3 cells were least sensitive to SeSp. Based on these results, we used LNCaP cells treated with the IC_50 _values of CSp and SeSp extracts for further experiments.

### PSA analysis

**Figure 3:**
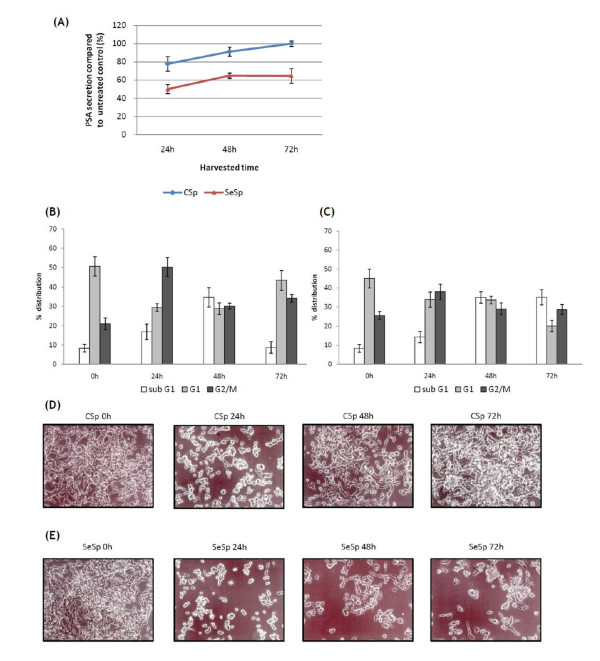
**Effects of 24 h treatment of CSp and SeSp to LNCaP cells**. Cells treated with CSp regained their normal PSA secretion, cell cycle distribution, and proliferation ability after being released from the CSp treatment. (A) PSA secretion in LNCaP cells treated with CSp and SeSp extracts. PSA values were normalized against the total protein concentration of viable cells. (B) Effects of CSp and (C) SeSp extracts on the LNCaP cell cycle. Cells were harvested at indicated times and analyzed by flow cytometry. (D) Representative photomicrographs of LNCaP cells treated with CSp and (E) SeSp extracts at each IC_50 _concentration at various time points.

The results of the PSA analysis are shown in Figure 3A. The PSA analysis was only performed with LNCaP cells, since PC-3 and DU-145 cells are androgen-independent and do not produce PSA [34]. LNCaP cells treated with both sprout extracts for 24 hours showed significantly decreased PSA secretion. However, after being released from the treatment, only cells treated with SeSp maintained the suppression of PSA secretion, while cells treated with CSp regained their normal PSA secretion rate.

### Flow cytometric analysis

To investigate whether both sprout extracts could induce cell cycle changes and cell death in prostate cancer cell lines, flow cytometric analysis of exponentially grown LNCaP cells treated with each IC_50 _was performed. As shown in Figures 3B and 3C, the induction of G2/M phase arrest upon treatment with both the CSp and SeSp extracts was noticeable as early as 24 hours after treatment, and reached a peak at 38.0-50.2%. These results suggest that the cells were undergoing cell death after prolonged mitotic blockage. By 48 hours after SeSp treatment, the number of sub-G1 (hypodiploid) cells was markedly increased, reaching peak levels at 72 hours (35.1%). By contrast, LNCaP cells treated with CSp demonstrated resistance to this phenomenon, since their cell cycle pattern tended to return to normal at 72 hours after treatment (Figure 3B). These results were similar to those of our PSA analysis (Figure 3A). The kinetics of the cell cycle distribution of cells treated with sprout extracts are shown in Figures 3B and 3C.

### Microscopic examination

When LNCaP prostate cancer cells were treated with CSp and SeSp extracts at each IC_50_, clear morphological changes were observed 24 hours after treatment (Figure 3D and 3E). Morphological changes in LNCaP cells were clearly observed 24 hours after treatment with both sprout extracts, and it was obvious that treatment with SeSp extracts induced cell death more effectively than CSp in LNCaP cells. This microscopic examination confirmed our results from the PSA and flow cytometric analyses, which indicated that after their release from 24 hours of CSp treatment, LNCaP cells regained their ability to proliferate and also seemed capable of evading apoptosis (Figure 3D). As anticipated, this phenomenon was not observed in cells treated with SeSp (Figure 3E).

### Western blot analysis of apoptotic-related proteins

**Figure 4:**
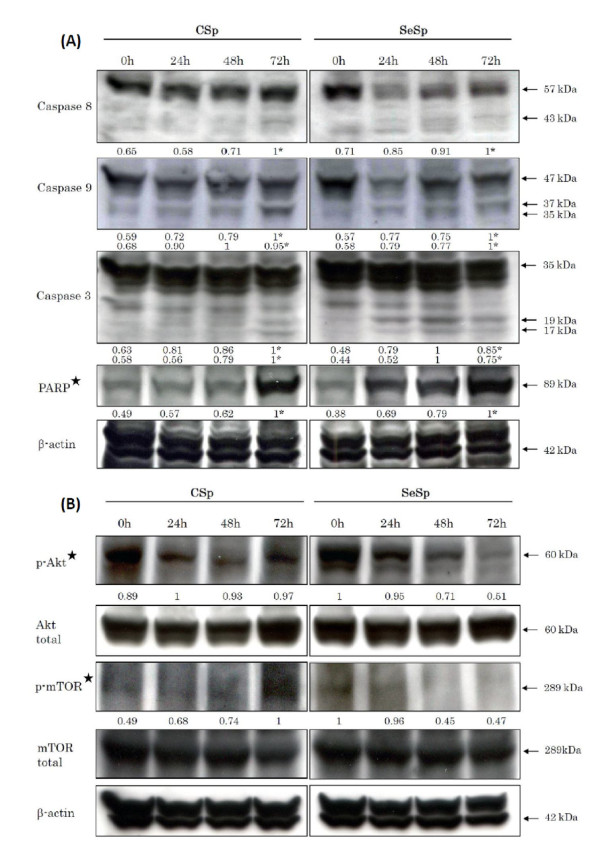
**Western blot analysis of (A) upregulation of the apoptotic-related proteins and (B) downregulation of cell survival-related proteins in LNCaP cells treated with CSp or SeSp for 24 h and harvested at the indicated times**. * indicates the cleaved form measured using densitometric analysis. ✮ indicates that the effects induced by CSp and SeSp are significantly different (*p *< 0.001, by two-way ANOVA).

Caspase signaling pathways, consisting of a death receptor-dependent extrinsic pathway and a death receptor-independent intrinsic pathway, were also examined in LNCaP cells treated with CSp and SeSp extracts at each IC_50_. The expression levels of active caspase-8 for the extrinsic pathway, caspase-9 for the intrinsic pathway, and caspase-3 were found to increase in LNCaP cells in a time-dependent manner (Figure 4A). The changes in the expression levels of poly (ADP-ribose) polymerase (PARP), described below, suggested that both sprout extracts induced apoptosis through both the extrinsic and intrinsic pathways. The expression levels of PARP, one of the best biomarkers of apoptosis, were analyzed within 24 hours of treatment with both sprout extracts. Result of two-way ANOVA analysis showed that the increased PARP expression induced by both extracts was significantly different (*p *< 0.001). The N-terminal fragment of PARP, which is an 89-kDa peptide cleaved from full-length PARP (116 kDa), was detected as early as 24 hours after SeSp treatment in LNCaP cells but at 72 hours in LNCaP cells treated with CSp (Figure 4A). These results suggest that SeSp induces apoptosis earlier than CSp in LNCaP cells.

### Western blot analysis of survival-related protein

As one of the most important survival signaling pathways in malignancy, the Akt/mTOR pathway has been reported to play an important role in determining the chemosensitivity of many kinds of cells [35-37]. We evaluated the expression of these survival signaling proteins in response to both sprout extracts in LNCaP cells. Figure 4B shows that SeSp inhibited the activation of Akt in LNCaP cells in a time-dependent manner, as shown by the reduced expression of phosphorylated Akt (p-Akt at Ser473). On the other hand, the expression of p-Akt in LNCap cells treated with CSp tended to increase within 72 hours. SeSp treatment also reduced the expression of phosphorylated mTOR (p-mTOR at Ser2448), which is the downstream target of Akt (Figure 4B). By contrast, CSp increased the presence of phosphorylated mTOR in LNCaP cells at 72 hours (Figure 4B). A two-way ANOVA then confirmed the effect of both extracts to these survival signaling protein expressions was significantly different (*p *< 0.001). These results indicate that the mTOR pathway might play an important role in maintaining the survival of LNCaP cells in response to CSp treatment. Previous *in vivo *studies have reported that sulforaphane itself as the active compound of broccoli sprouts was able to inhibit Akt activation of prostate tumor tissues [16]; however, it required a much higher dose and longer treatment period than what we used in the present study. Taken together, our results suggest that, unlike CSp, SeSp downregulates the Akt/mTOR pathway of prostate cancer LNCaP cells.

## Discussion

Our results suggest that in the SeSp extract, Se accumulates in the form of Se-methylselenocysteine. This result is in agreement with those of Sugihara and co-workers [27] who reported that Se-methylselenocysteine was the major Se compound that accumulated when broccoli sprout seeds germinated in an inorganic Se solution. Plants primarily take up inorganic Se, which is then metabolized into several Se analogs of various sulfur metabolites via the sulfur assimilation pathway [17]. Although Se toxicity in plants is thought to be caused by the nonspecific binding of seleno-amino acids to proteins [38], plants are able to convert the potentially toxic seleno-amino acids into non-protein derivatives such as Se-methylselenocysteine [39]. Se-methylselenocysteine is a mono-methylated Se and is a precursor of methylselenol, a critical metabolite in Se chemoprevention [5,20]. Se-methylselenocysteine can directly enter the methylated pool and be converted to methylselenol by a lyase-catalyzed reaction [5,20,40]. It has been reported that Se-methylselenocysteine is able to inhibit the growth of many types of cancer cells [19,41-43].

Broccoli sprouts have been reported to inhibit skin and urinary bladder carcinogenesis *in vivo *[24,25] and also inhibit the proliferation of human bladder and prostate cancer cells *in vitro *[23,44]. The anticancer properties of broccoli sprouts occur through their primary active micronutrient, sulforaphane [45], by the induction of mitochondria-mediated apoptosis [23], induction of cycle arrest [23,45], inhibition of histone deacetylase (HDAC) [46], and the induction of phase 2 enzymes, including glutathione S-transferase (GST) and quinone oxidoreductase 1 (NQO1) [47,48]. Moreover, a recent clinical study from the UK suggested that broccoli sprouts also interact with the glutathione S-transferase mu 1 (GSTM1) genotype to disrupt oncogenic signaling pathways [49].

In this study, we have shown that broccoli sprout extracts inhibit the growth of prostate cancer cell lines in a dose-dependent manner. Furthermore, the addition of Se enhances the antiproliferative effects of broccoli sprouts. In all three prostate cancer cell lines tested, SeSp was superior to CSp in inhibiting cell proliferation (Figure 2) and the IC_50 _of SeSp was almost two-fold lower than that of CSp. Growth inhibition was accompanied by the suppression of PSA secretion, a well-accepted biomarker for the diagnosis and prognosis of prostate disease [50], of androgen-dependent LNCaP cells.

The induction of apoptosis and the inhibition of survival signaling pathways in cancer cells are the two main goals in cancer treatment [37]. Here, we provide evidence that the inhibition of carcinogenesis by SeSp was mediated through the regulation of multiple signaling pathways. Our results suggest that broccoli sprouts induce a G2/M-phase arrest in prostate cancer cell lines that is accompanied by an increase in sub-G1 cells, which represent a cell death population. Western blot analysis of LNCaP cells was then performed to confirm apoptotic cell death. Cell death was found to be initiated extrinsically via the caspase-8 pathway, intrinsically via the mitochondrial caspase-9 pathway, and also by caspase-3 and PARP activation. In addition, SeSp was found to downregulate the Akt/mTOR pathway by decreasing p-Akt expression and the downstream expression of p-mTOR. Downregulation of these survival signaling pathways was found to enhance the ability of apoptosis-related proteins to initiate the apoptosis machinery [37] and therefore should be beneficial for the prevention and treatment of malignant diseases. Interestingly, the downregulation of the Akt/mTOR pathway was only observed for SeSp treatment, not for CSp treatment. In fact, after LNCaP cells were released from CSp treatment, the expression of p-mTOR in treated cells tended to increase. This result might explain the reason that LNCaP cells escaped apoptosis and regained their normal proliferation ability after release from CSp treatment.

**Figure 5:**
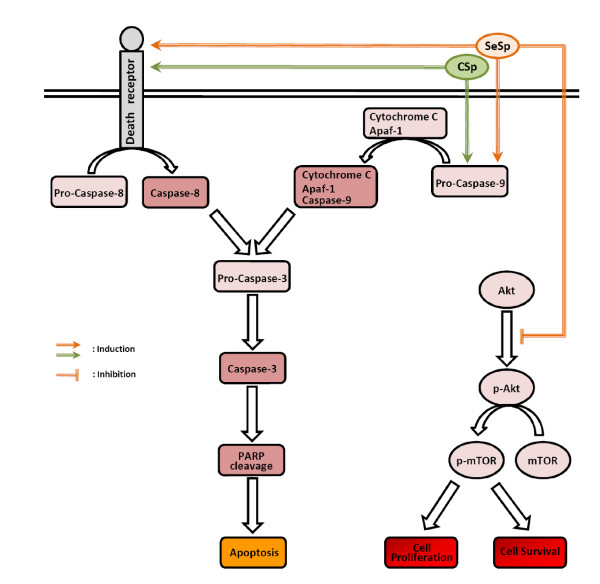
**Proposed schematic charts showing various mechanisms by which CSp and SeSp extracts inhibit LNCaP cell proliferation**. Both extracts induced apoptosis through the extrinsic caspase-8 and intrinsic caspase-9 pathway. However, only the SeSp extract inhibited Akt phosphorylation and downstream mTOR phosphorylation, resulting in the inhibition of cell proliferation and survival.

A growing body of evidence suggests that products derived from plants are useful in the prevention and treatment of cancer. Thus, understanding the mechanisms of action of such products may provide valuable information for their possible application in cancer prevention and therapy. The anticancer activity of broccoli sprouts was first introduced by Fahey et al. [51] in 1997, when they reported its ability to protect rats from chemically-induced mammary tumor development. In 2001, Finley et al. [52] introduced the idea of Se enrichment to broccoli sprouts to extend their protective role against cancer. In the present study, we showed that SeSp inhibits cancer cell proliferation by several different mechanisms (Figure 5). Here, we have proposed that Se enrichment enhances the cancer cell proliferation inhibition and apoptosis induction of CSp. Moreover, Se also decreases the risk of cancer recurrence after release from CSp treatment.

## Conclusion

Se is a well-known anticancer agent [5], and broccoli sprouts are commonly consumed vegetables with anticancer properties [51,52]. Thus, although further studies related to their safety will be necessary, Se-enriched broccoli sprouts have potential as a chemopreventive nutraceutical for prostate cancer management. The anticancer properties of SeSp extract are currently being evaluated in our laboratory with a series of different cancer cell lines, as well as through *in vivo *experiments using an animal model.

## Competing interests

The authors declare that they have no competing interests.

## Authors' contributions

RA carried out molecular, cell biology, and data analysis, participated in the study design and drafted the manuscript. AF, KK, CY and EWR participated in molecular, cell biology and data analysis. KI, KS, MM, HKu and HKo conceived of the study and participated in its design and coordination. All authors read and approved the final manuscript.

## Pre-publication history

The pre-publication history for this paper can be accessed here:

http://www.biomedcentral.com/1471-2407/9/414/prepub

## Supplementary Material

Additional file 1 **Instrumental operating conditions for Se speciation of Se-enriched broccoli sprouts**. The data provided the instrumental operating condition of HPLC/ICP-MS for Se speciation. Click here for file

Additional file 2 **Total Se concentration, Se compounds concentration, and sulforaphane concentration of the sprout extracts**. The data provided the total Se concentration, Se compounds concentration, and sulforaphane concentration of the control and Se-enriched sprout extracts. Click here for file

Additional file 3 **IC_50 _concentration of Sulforaphane (SF) and Se-methylselenocysteine (Se-MSC) in CSp and SeSp on LNCaP, PC-3, DU-145, and CHEK-1 cells**. The data provided the concentration of sulforaphane and Se-methylselenocysteine at the IC_50 _dilution of CSp and SeSp extracts on each cells. Click here for file
