# Supplementary material for: Selenium enrichment of broccoli sprout extract increases chemosensitivity and apoptosis of LNCaP prostate cancer cells
Source: BMC Cancer. 2009 Nov 30;9:414. doi: 10.1186/1471-2407-9-414 (PMC2794877; doi:10.1186/1471-2407-9-414)
Supplement: Additional file 1 — Instrumental operating conditions for Se speciation of Se-enriched broccoli sprouts. The data provided the instrumental operating condition of HPLC/ICP-MS for Se speciation. [file 1471-2407-9-414-S1.DOC]

**Additional Files:**

Additional File 1. Instrumental operating conditions for Se speciation of Se-enriched broccoli sprouts

| Chromatographic system: | |  |
| --- | --- | --- |
|  | HPLC column | Asahipak GS-320 HQ |
|  |  | (300 x 7.6 mm i.d.), 6 μm |
|  | Mobile phase | 20 mM Potassium phosphate buffer |
|  |  | at pH 6.8 |
|  | Flow rate | 0.6 ml/min |
|  | Sample injection volume | 50 μl |
|  |  |  |
| ICP-MS: | |  |
|  | Forward power | 1400 W |
|  | Lens voltage | 6.25 volts |
|  | Dwell time | 100 ms |
|  | Nebulizer gas flow | 1.02 l/min |
|  | Plasma gas flow | 15 l/min |
|  | Isotopes monitored | ^77^Se and ^82^Se |
