# Supplementary material for: Selenium enrichment of broccoli sprout extract increases chemosensitivity and apoptosis of LNCaP prostate cancer cells
Source: BMC Cancer. 2009 Nov 30;9:414. doi: 10.1186/1471-2407-9-414 (PMC2794877; doi:10.1186/1471-2407-9-414)
Supplement: Additional file 2 — Total Se concentration, Se compounds concentration, and sulforaphane concentration of the sprout extracts. The data provided the total Se concentration, Se compounds concentration, and sulforaphane concentration of the control and Se-enriched sprout extracts. [file 1471-2407-9-414-S2.DOC]

Additional File 2. Total Se concentration, Se compounds concentration, and sulforaphane concentration of the sprout extracts

|  |  | Control sprouts | Se-enriched sprouts |
| --- | --- | --- | --- |
| Total selenium (μg/g) | | 0.01±0.008 | 10.24±1.54* |
| Se speciation: | |  |  |
|  | Se-methylselenocysteine (μM) | nd | 24.20±0.85 |
|  | Selenite (μM) | nd | 6.16±0.28 |
|  | Selenate (μM) | nd | 2.00±0.07 |
| Sulforaphane (μM) | | 375.87±17.98 | 316.53±43.54 |

nd: not detected

*: *p* < 0.05
