# Supplementary material for: Selenium enrichment of broccoli sprout extract increases chemosensitivity and apoptosis of LNCaP prostate cancer cells
Source: BMC Cancer. 2009 Nov 30;9:414. doi: 10.1186/1471-2407-9-414 (PMC2794877; doi:10.1186/1471-2407-9-414)
Supplement: Additional file 3 — IC50 concentration of Sulforaphane (SF) and Se-methylselenocysteine (Se-MSC) in CSp and SeSp on LNCaP, PC-3, DU-145, and CHEK-1 cells. The data provided the concentration of sulforaphane and Se-methylselenocysteine at the IC50 dilution of CSp and SeSp extracts on each cells. [file 1471-2407-9-414-S3.DOC]

Additional File 3. IC_50_ concentration of Sulforaphane (SF) and Se-methylselenocysteine

(Se-MSC) in CSp and SeSp on LNCaP, PC-3, DU-145, and CHEK-1 cells

|  | CSp treatment | | SeSp treatment | |
| --- | --- | --- | --- | --- |
|  | SF (μM) | Se-MSC (μM) | SF (μM) | Se-MSC (μM) |
| LNCaP | 19.8 | nd | 9.9 | 0.8 |
| PC-3 | 18.8 | nd | 11.3 | 0.9 |
| DU-145 | 25.0 | nd | 10.5 | 0.8 |
| CHEK-1 | 53.7 | nd | 22.6 | 1.7 |

nd: not detected
